# The Effect of Intervention Model Education on the Quality of Life of Hypertensive Patients: A Quasi-Experimental Study

**DOI:** 10.34172/jrhs.9141

**Published:** 2025-09-15

**Authors:** Agustina Boru Gultom, Arbani Batubara

**Affiliations:** ^1^Nursing Department, Politeknik Kesehatan Kementerian Kesehatan Medan, North Sumatra, Indonesia

**Keywords:** Quality of life, Hypertensive patients, Model, Educational intervention

## Abstract

**Background:**

The increasing prevalence of hypertension (HTN), accompanied by a decreasing quality of life (QoL), requires appropriate interventions to avoid its impacts and the occurrence of chronic conditions. The purpose of this study was to assess the effect of the intervention model education on the QoL of hypertensive patients compared to HTN self-management training and no intervention.

**Study Design:**

A quasi-experimental study.

**Methods:**

The sample consisted of 138 hypertensive patients, divided into an HTN intervention model education group (n=46), an HTN management training group (n=46), and a group without treatment (n=46). The study used a quasi-experimental design with a control group. All groups received a pre-test, and after 6 weeks, they all received a post-test with the WHOQOL-BREF questionnaire.

**Results:**

The HTN intervention model education group and the HTN self-management training group had a significant effect on the QoL of hypertensive patients (*P*=0.0001), while the control group showed no effect (*P*=0.310). The Kruskal-Wallis test demonstrated a significant difference in the three interventions, and the highest difference was observed in the HTN intervention model education group.

**Conclusion:**

The HTN intervention model education was the main choice because it involved not only the patient himself but also policies, health workers, cadres, and families, as well as the presence of booklets.

## Background

 Hypertension (HTN) is a global public health emergency, with a decline in the quality of life (QoL) of patients.^1^ The magnitude of the problem is related to the increasing prevalence of HTN in the world and Southeast Asia, and the percentage of controlled HTN is still low. In addition, stroke complications and cardiovascular disorders exist, premature death has emerged,^2^ and the QoL of hypertensive patients is still at a slightly low level.^1,3^

 HTN affects the QoL of patients.^4^ The QoL of patients with HTN related to health is low in all domains of QoL,^1^ especially in terms of pain or discomfort because of the influence of high blood pressure.^5,6^ QoL scores in hypertensive women are lower than in men in all physical, psychological, social, and environmental dimensions.^7^ The older a woman with HTN is, the lower her QoL will be compared to men.^8,9^

 The low QoL of hypertensive patients can result in various complications. Hypertensive emergency, especially when complications have reached end-organ damage (i.e., in acute-chronic kidney disease), will have an impact on increasing mortality rates.^10^

 Self-management is one of the dominant factors that can improve the QoL of hypertensive patients.^11^ Self-management is more effective than medical care in treating patients with chronic diseases because it can increase self-efficacy, reduce symptoms of depression, adapt to changes caused by the disease, manage the disease itself, and improve the patient’s QoL.^12^ However, based on the results of previous studies, not all self-management interventions have a positive correlation with the QoL of hypertensive patients.^13,14^ Regardless of self-management, there are still interventions that can improve the QoL of hypertensive patients, such as family support or social support. Based on previous studies, this approach has shown a significant impact, namely the continuous care model, by including family members in patient care.^15^ However, some do not show significant results, such as the HTN management model, which includes the design of a patient delivery system, support in decision-making, clinical information systems, and self-management support.^16^

 The socio-ecological model (SEM) has the potential to create better conditions than just looking at individual behavior by considering various social and cultural influences in the development of community behavior.^17^ Good HTN care is accessible and strengthens primary health care. The challenge now is to move from affordable care to attainable care This is because treating HTN through primary health care will save lives while saving billions of dollars annually.^18^

 The intervention model of QoL of hypertensive patients needs to be designed based on the modification of the SEM. Therefore, this study aims to analyze the influence of education on the HTN QoL intervention model when compared with HTN self-management or no treatment.

## Methods

###  Participant characteristics and research design

 The research method was quantitative with a quasi-experimental design, and the participants of this study were assigned to an HTN intervention model group, an HTN self-management training group, and a control group. The research was conducted in Deli Serdang Regency, North Sumatra Province, Indonesia, in May-October 2024. Deli Serdang Regency was selected based on the prevalence of HTN in this regency, which exceeded that of North Sumatra Province.^19^ The study population was all female hypertensive patients who were treated in the working areas of Mulyorejo, Kutalimbaru, and Sei Semayang Health Centers, Deli Serdang Regency.

 This study used a sample of female hypertensive patients who met the inclusion criteria, including women aged 18–70 years who had HTN and consumed antihypertensive drugs. Consecutive sampling techniques were used for sampling. Prospective participants who agreed to participate in the research activities signed the consent form. The single sample size formula in hypothesis testing was considered for sample collection using the correlation coefficient (r). The desired confidence level was 99% with α = 0.05, so that zα = 1.960. The research power was set at 90% so that zβ = 1.282. The R value was an estimate of the existing correlation coefficient obtained from literature studies. In the previous study, the correlation coefficient of self-care behavior with the QoL of HTN sufferers was 0.196.^20^ From the calculation results, each group had 46 samples, and the total sample was 138 respondents. The sampling technique was consecutive sampling, with the sample criteria being those with HTN, women, using at least 1 type of HTN medication, being able to communicate using good Indonesian, being ≥ 18 years old, and willing to be respondents.

###  Intervention

 The HTN intervention model education treatment group included 4 small groups in four locations in the Mulyorejo Health Center working area, Deli Serdang Regency. Each small group received 1 day of pre-testing with the World Health Organization Quality of Life-Brief Version (WHOQOL-BREF) questionnaire and 1 day of intervention model education with the involvement of cadres and patient families.

 This questionnaire consists of 26 statements. Two statements measure overall and general health QoL, and the remaining 24 questions are divided into four domains, including physical, psychological, and social relationships, and the environment. Each statement was rated on a scale of 1–5. The scores were then converted linearly to a scale of 0–100. The Indonesian version of the WHOQOL-BREF questionnaire was valid and reliable for use in Indonesia.^21^ The cutoff value of the WHOQOLBREF scale is 71.5 points. Values < 71.5 indicate poor QoL, while values equal to 71.5 and > 71.5 represent good QoL.^22^

 The hypertensive patient QoL intervention model included a combination of research results on factors related to the QoL of hypertensive patients. These factors included stress, physical activity, salt consumption, blood pressure checks, blood pressure, body mass index (BMI), comorbidities, consumption of antihypertensive drugs, family support, and self-efficacy developed in one booklet combined with a SEM. This model involved various health workers at the health center, including activity permits through the head of the health center an education team including doctors, non-communicable diseases, nutrition, nurses, public health, and the elderly through focus group discussions. The education team met and agreed on a policy for the HTN intervention model that was implemented, the criteria for the participant sample used as the intervention model, the number of groups that were intervened, the location where the model was implemented in four locations, health workers who acted as evaluators, and cadres who acted as monitors of patients and families. They also discussed and agreed on the selection of intervention model education topics to improve the QoL of hypertensive patients for the health worker team, the time and place of implementation of education at each location of the intervention model implementation, and the agreed-upon hypertensive patient QoL education module for educators and cadres. The HTN intervention model education module contained twelve chapters covering an overview of HTN, HTN and QoL, blood pressure, blood pressure checks, comorbidities, regularity of antihypertensive drug consumption, family support, BMI, physical activity, salt consumption, stress, and self-efficacy. The intervention model education for HTN involved health workers (i.e., doctors, non-communicable disease specialists, nutritionists, nurses, and public health workers) and the elderly who had agreed on the model and module used through focus group discussions. The methods utilized in the HTN intervention model education were lectures, discussions, questions and answers, demonstrations, and redemonstrations on progressive muscle relaxation techniques, accompanied by classical music using speakers. Each participant received a booklet on developing the QoL of hypertensive patients in the intervention model education activity on the QoL of hypertensive patients. The next day belonged to monitoring patient behavior in developing the QoL of HTN and their families every day for six weeks until the post-test was performed again. Cadres monitored patient behavior in improving the QoL of HTN with their families through a format. Meanwhile, health workers in the non-communicable disease section provided guidance to cadres, performed evaluation activities on the performance of each cadre, and reported the data in a format. After six weeks, health workers in the noncommunicable diseases section, together with researchers, conducted a post-test of research on QoL with the same questionnaire as the one used during the pre-test.

 The HTN self-management training treatment group included 4 small groups in four locations in the Kutalimbaru Health Center work area, Deli Serdang Regency. Each small group received 1 day of pre-test QoL with the WHOQOL-BREF questionnaire and 1 day of HTN self-management training using the HTN self-management booklet. The booklet contained information about HTN self-management behavior with an emphasis on nursing based on the development of literature reviews and previous research. The training began with the provision of information about the description of HTN, followed by the theme of self-management behavior, including blood pressure checks, consumption of antihypertensive drugs, maintaining body weight (BMI) with a healthy diet, doing physical activity, consuming salt, and managing stress. The following day, participants were monitored at their respective homes. The booklet provided during the training was a guide for participants in doing HTN self-management behavior independently at home. After six weeks of training, the researchers conducted a post-test on QoL using the same questionnaire.

**Figure 1:**
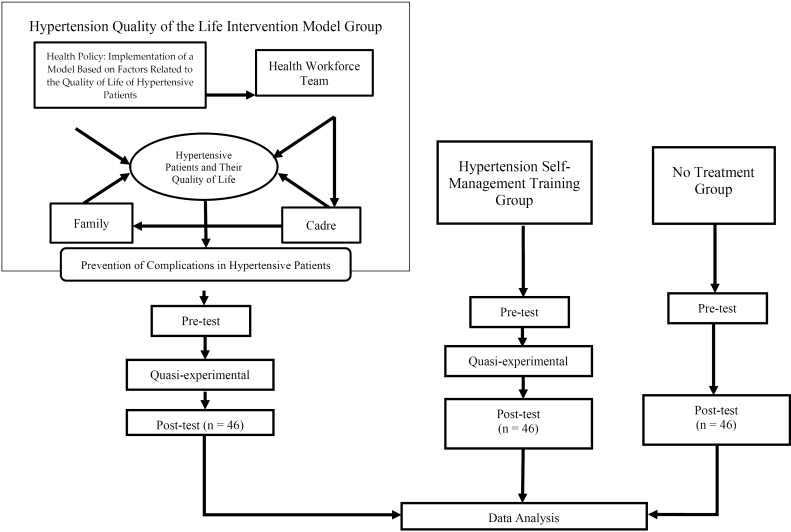
The research chart

 The control group included 4 small groups in four locations in the working area of the Sei Semayang Health Center, Deli Serdang Regency. Each small group received 1 day of pre-testing on QoL with the WHOQOL-BREF questionnaire. Then, after six weeks, the researchers performed a post-test on each small group with the same questionnaire. Figure 1 shows the research chart.

###  Data analysis

 This study considered quantitative data analysis techniques, with a paired t-test and Kruskal-Wallis test for bivariate and multivariate analyses, respectively, using IBM Statistical Package and Service Solution (SPSS) software (version 22.0) for Windows. The significance was utilized when the *P *value was ≤ 0.05, with a 95% confidence interval.

## Results

 The results (Table 1) demonstrated that all hypertensive participants were female in the control, self-management, and model groups, each totaling 46 respondents (100%). Based on age, most participants of the control (n = 24, 52.2%) and self-management (n = 27, 58.7%) groups were 51–60 years old and 61–70 years old, respectively. Moreover, 20 participants of the model group (43.5%) were 51–60 years old. As regards marriage, 78.3%, 67.4%, and 65.2% of the participants of the control (n = 36), self-management (n = 31), and model (n = 30) groups were married, respectively. Concerning education, 20 (43.5%), 24 (52.2%), and 21 (45.7%) respondents in the control, self-management, and model groups were at the high, elementary, and elementary school levels, respectively. In terms of occupation, the majority of participants in the control (n = 39, 84.8%), self-management (n = 28, 60.9%), and model (n = 41, 89.1%) groups were housewives, farmers, and housewives, respectively.

**Table 1:** Characteristics of hypertensive participants in the control, self-management, and model groups

**Characteristics**	**Control**		**Self-Management**		**Model**	
	**Frequency**	**Percent**	**Frequency**	**Percent**	**Frequency**	**Percent**
Age (year)						
31-40	0	0.0	0	0.0	2	4.3
41-50	12	26.1	7	15.2	7	15.2
51-60	24	52.2	12	26.1	20	43.5
61-70	10	21.7	27	58.7	17	37
Marital status						
Not married yet	2	4.3	1	2.2	0	0.0
Married	36	78.3	31	67.4	30	65.2
Widow	8	17.4	14	30.4	16	34.8
Education						
Elementary school	14	30.4	24	52.2	21	45.7
Junior high school	8	17.4	16	34.8	11	23.9
Senior high school	20	43.5	3	6.5	14	30.4
College	4	8.7	3	6.5	0	0.0
Type of work						
Housewife	39	84.8	14	30.4	41	89.1
Government employees	1	2.2	0	0.0	0	0.0
Teacher	1	2.2	2	4.3	0	0.0
Farmer	1	2.2	28	60.9	1	2.2
Retired	4	8.7	1	2.2	0	0.0
Self-employed	0	0.0	1	2.2	1	2.2
Private employees	0	0.0	0	0.0	3	6.5

Table 1 presents the characteristics of the participants. Based on the results (Table 2), the largest mean of the control group before and after the intervention belonged to the QoL in terms of psychology. Similarly, the largest mean of the self-management training and model education groups before and after the intervention was related to the QoL in terms of psychology.

**Table 2:** Minimum, maximum, mean, and standard deviation scores of the quality of life of the control, self-management, and model groups

**Variables**	**Minimum**	**Maximum**	**Mean**	**SD**
Control group				
Physical aspects before the intervention	31	81	61.17	13.63
Psychological aspects before the intervention	31	94	68.00	15.45
Social aspects before the intervention	22	75	55.30	14.06
Environmental aspects before the intervention	38	81	59.59	11.86
Physical aspects after the intervention	31	81	61.61	13.31
Psychological aspects after the intervention	31	94	67.57	14.57
Social aspects after the intervention	22	75	56.26	14.97
Environmental aspects after the intervention	44	81	61.09	12.11
Quality of life (QoL) before the intervention	33	81	61.15	11.59
QoL after the intervention	33	81	61.72	11.65
Self-management group				
Physical aspects before the intervention	25	75	54.98	10.53
Psychological aspects before the intervention	19	88	57.30	15.64
Social aspects before the intervention	25	81	51.24	11.09
Environmental aspects before the intervention	19	75	52.37	12.46
Physical aspects after the intervention	38	88	64.46	10.12
Psychological aspects after the intervention	31	94	69.22	13.56
Social aspects after the intervention	31	81	59.72	11.07
Environmental aspects after the intervention	25	81	59.07	11.49
QoL before the intervention	36	70	53.87	8.96
QoL after the intervention	47	78	63.22	8.48
Model group				
Physical aspects before the intervention	38	88	60.57	9.76
Psychological aspects before the intervention	38	88	63.26	12.17
Social aspects before the intervention	31	75	56.43	12.57
Environmental aspects before the intervention	44	88	60.28	10.94
Physical aspects after the intervention	38	88	64.35	9.95
Psychological aspects after the intervention	44	88	72.41	10.50
Social aspects after the intervention	44	94	62.59	12.30
Environmental aspects after the intervention	44	94	64.52	9.90
QoL before the intervention	44	77	60.26	8.50
QoL after the intervention	50	88	69.85	8.51

*Note*. SD: Standard deviation.

 According to the findings (Table 3), the largest difference in the mean QoL scores was in the intervention model education activity, although the *P* value was 0.0001 in both the intervention model education group and the HTN self-management group.

**Table 3:** Effect of intervention model education, hypertension management training, and lack of treatment/control on the quality of life of hypertensive patients

**Treatment Effect**	**Mean Difference**	***P*** ** value**
Quality of life (QoL) before and after intervention model education	9.59	0.001
Physical dimension before and after the education intervention model	3.78	0.001
Psychological dimensions of the QoL before and after the educational intervention model	9.15	0.001
Social dimensions of the QoL before and after the educational intervention model	6.15	0.001
Environmental dimensions of the QoL before and after the educational intervention model	4.24	0.002
QoL before and after hypertension (HTN) self-management training	6.59	0.001
Physical dimensions of the QoL before and after HTN self-management training	7.15	0.001
Psychological dimensions of the QoL before and after HTN self-management training	9.33	0.001
Social dimensions of the QoL before and after HTN self-management training	4.83	0.001
Environmental dimensions of the QoL before and after HTN self-management training	4.67	0.001
QoL before and after no treatment	0.57	0.310
Physical dimensions of the QoL before and after no treatment	0.43	0.541
Psychological dimensions of the QoL before and after no treatment	0.43	0.707
Social dimensions of the QoL before and after no treatment	1.50	0.065
Environmental dimensions of the QoL before and after no treatment	0.57	0.310

 The results of the multivariate analysis, which aimed to analyze the differences in the QoL scores after activities in the intervention model education, HTN self-management training, and control groups, are provided in Table 4. The Kruskal-Wallis test was employed because the results of the normality test showed that the data distribution was not normal. The results of the statistical test (*P*-value) were 0.0001, demonstrating that there was a significant difference in the QoL in all types of interventions, and the best type of intervention to improve the QoL for HTN was through education on the HTN intervention model.

**Table 4:** Differences in the quality of life in the control group, hypertension self-management training group, and intervention model education group

**Types of interventions for hypertension quality of life**	**Mean rank**	***P*** ** value**
No treatment	59.01	0.001
Hypertension self-management training	61.20	
Hypertension intervention model education	88.29	

## Discussion

 This study investigated the effectiveness of education on the HTN QoL intervention model when compared with self-management of HTN or without treatment.

 The characteristics of female respondents with HTN represented no striking differences in the HTN intervention model education, the HTN self-management training, and the control groups, although there were slight differences among the three groups. In terms of age, the control and model groups were mostly aged 51–60 years, while participants of the self-management group were in the age range of 61–70 years. The three groups were mostly married. In addition, the model and self-management groups were dominated by elementary school education, while the control group was dominated by high school education. Most participants of the control and model groups were housewives, while those of the self-management group were farmers. Based on the duration of HTN, the three groups mostly experienced it for less than 3 years.

 The physical health dimension of QoL included activities of daily living, dependence on medication, energy level, mobility, pain, sleep, and capacity to work ^23^. The physical dimension score of QoL before and after the intervention in the three groups showed that it was still below 71.5, or still poor. However, action in the HTN QoL intervention model group and the HTN self-management training group had a significant effect on the physical dimension. Physical activity programs in primary care provided to hypertensive patients improve heart and blood vessel health, support blood control efforts, and enhance QoL.^24^ The HTN QoL intervention model group and the HTN self-management training group received a physical activity program package, the only difference being that the HTN QoL intervention model group received support from family, cadres, and health workers. However, in reality, the difference in the mean score of the physical dimension related to the QoL of the HTN QoL intervention model group was smaller than that of the HTN self-management training group. Previous studies reported that sociodemographic characteristics of hypertensive patients affect their QoL, considering that hypertensive patients with comorbidities, such as diabetes and obesity, tend to have a lower QoL,^25,26^ low educational status, housewife, low income,^27^ and others. Nonetheless, further studies are needed in this regard.

 The psychological dimension included body image, negative feelings, positive feelings, self-esteem, spirituality, and cognition. The highest score increase and the highest mean difference were observed in the psychological domain in both intervention groups. Significant results were found in the psychological domain in the HTN QoL intervention model and HTN self-management training groups. Psychological interventions can serve as non-drug therapies that help patients understand the psychological and social factors involved in HTN and the important role of medication in stabilizing blood pressure and limiting adverse health effects. Moreover, they help patients improve behavior, improve interpersonal communication, and enhance social functioning, thus positively affecting social relationships, which is another dimension of QoL.^28^

 The social relationship dimension encompassed personal relationships, social support, and sex life. The social dimension scores of QoL before and after the intervention in the three groups were still below 71.5 or still poor. Action in the HTN QoL intervention model group and the HTN self-management training group had a significant effect on the social domain. Improving the QoL of hypertensive patients is related to the presence of social support^29^ and family function.^30^ To manage hypertensive patients, the role of community health workers is necessary.^31^ The mean difference in the social dimension was greater in the HTN QoL intervention model group when compared to the HTN self-management training group because the HTN QoL intervention model group included support from various groups, such as family, cadres, and health workers.

 The environmental dimension consisted of financial resources, safety, access to health and social services, home environment, opportunities to acquire new skills and knowledge, recreation, transportation, and the physical environment. The environmental dimension scores of QoL before and after the intervention in the three groups were still < 71.5, or still poor. Action in the HTN QoL intervention model group and the HTN self-management training group could noticeably influence the environmental domain of the QoL. Interventions regarding the impact of the environment on blood pressure in hypertensive patients are associated with behavioral modifications (e.g., eating habits caused by environmental factors, obesity, and stress) that can affect cardiovascular health.^32,33^

 The mean difference in the QoL of hypertensive patients varied in the three types of treatment. The largest difference was found in the HTN QoL intervention model group, followed by the HTN self-management training group. However, the smallest mean difference belonged to the group without treatment. Of the three types of intervention, two types had a significant effect on the QoL of hypertensive patients, including HTN intervention model education treatment and HTN self-management training. Good training provides an opportunity for self-acceptance and growth, facilitates thought flow, increases self-esteem and happiness of individuals and groups, and leads to sustainable well-being.^34^

 Conversely, lack of treatment did not have a significant effect. The group of female patients with HTN who consumed antihypertensive drugs but did not receive other interventions still showed an increase in their QoL, although small, by 0.57. This implies that a female patient with HTN can maintain their QoL or experience a slow increase by only using antihypertensive drugs. Previous studies demonstrated that respondents with medication adherence alone without other interventions are well associated with health-related QoL.^35^ However, the use of antihypertensive drugs did not cause changes in the overall QoL of patients and only had a positive effect on physical and mental aspects.^36^

 Nonetheless, the *P* value of QoL in the HTN QoL intervention model group was the same as that of the HTN self-management training group (*P* = 0.0001). The largest difference in the mean score of QoL was in the HTN QoL intervention model group (*P* = 9.59) compared to the HTN self-management training group (*P* = 6.59). This was also reinforced by the difference test with the Kruskal-Wallis test, demonstrating significant differences in the three groups, and the HTN QoL intervention model group was most able to improve QoL based on the mean rank.

 The implementation of the HTN QoL intervention model not only involves patients but also the Community Health Center institution as a policymaker and the community, including health workers, cadres, and families. Institutions, communities, and policies can encourage the success of efforts to modify the social and political environments to improve health. This is an investment in a highly important action in addressing the challenges associated with having a chronic disease.^37,38^ Health promotion uses principles, including the body of health knowledge, concepts, and theories derived from research results. The method, through empowering health communities, aims to develop the capacity of participants in making decisions, which refers to counseling, increasing community growth, and using social learning theory that emphasizes a strong emphasis on certain learning methods.^39^ There were many components in health promotion, but the most important was to encourage community participation and make it the property of the community.^40^

 The use of booklets aimed to convey information about self-behavior in controlling HTN to respondents by designing it as attractively as possible. The right media and social marketing methods in health campaigns provide real benefits in combating disease problems and bad habits.^41^

 Previous studies have reported significant results in the use of booklets or similar tools. The use of booklets showed noticeable results in motivating individuals to change habits in improving bone health.^42^ The booklet for developing the QoL of hypertensive patients has begun with a survey of factors related to the QoL of hypertensive patients, including stress, physical activity, salt consumption, blood pressure checks, blood pressure, body mass index, comorbidities, consumption of antihypertensive drugs, family support, and self-efficacy.^43^ After obtaining these factors, interventions were developed to address each related factor through the latest theories in nursing and health. The booklet contained material on what HTN is, what impact HTN has on QoL, and how to improve the QoL of hypertensive patients. The booklet on developing the QoL of hypertensive patients has received good input and positive validation from promotion and material experts in the health field. Then, this booklet had also received responses from respondents regarding the preparation of the booklet, the process of writing the booklet, the aesthetic aspects of the booklet, and the motivational aspects of the booklet, indicating good responses from respondents. In general, it revealed that the booklet can be used as a health promotion tool to improve the behavior and QoL of hypertensive patients.

 The intervention model for the QoL of hypertensive patients was a combination of factors related to the QoL of hypertensive patients with a SEM. The SEM provided a framework for understanding the interaction of important factors at the individual, relationship, community, and social levels that affect health.^44^ This model utilized principles in addressing challenges associated with religious, cultural, or political considerations. Effective implementation of this approach has the potential to improve the recruitment and retention of hard-to-reach populations. The use of the SEM lays the foundation for effective, sustainable success. Therefore, this model can be adapted to both developing and developed regions.^45^ This model has engaged the community more effectively and provided an opportunity to express it in action by considering various influences from the environment. The core of community engagement activities was the recognition and incorporation of the socio-cultural environment. Consideration of various influential impacts (e.g., economic, technological, political, physical, and individual impacts) is important to ensure that community engagement activities are complex and consider the social and structural determinants of health.^17^

 There were some limitations in this study. Participant similarity was controlled only through female gender, a diagnosis of HTN, a history of using at least one type of antihypertensive drug, and age ≥ 18 years. This study could not control other characteristics, such as the duration of HTN, the presence or absence of comorbidities, and the same QoL scores before the intervention in each group. The long time interval between before and after the research intervention provides an opportunity for more improvements in QoL scores; nonetheless, this study lasted only six weeks. Accordingly, it is recommended that future researchers control sample selection and extend the period before and after the intervention to 3–6 months.

**Highlights**

- The hypertension (HTN) intervention education model had a better effect on improving the quality of life (QoL) of hypertensive patients compared to HTN self-management training.
- The HTN intervention education model involved patients, policies, health workers, cadres, families, and booklets.
- Education using the socio-ecological model (SEM) was effective in improving the QoL of patients in each domain.

## Conclusion

 The results of this study confirmed a significant difference in improving the QoL of women with HTN using the HTN QoL intervention model compared to HTN self-management training and no treatment. The HTN QoL intervention model was a combination of factors related to the QoL of hypertensive patients, and the SEM was used to improve these patients’ QoL. The booklet for developing the QoL of HTN patients received good guidance and validation from health promotion and material experts and good responses from female respondents with HTN to support the implementation of the HTN patient QoL intervention model.

## Acknowledgements

 We express our sincere gratitude to all those who helped us in completing this research. Special thanks go to the Directorate of Health Polytechnic of the Ministry of Health Medan and the Nursing Department for their support during the research. We also appreciate the contributions of our Community Health Center partners, friends, and family members, who provided encouragement and support during the research process.

## Competing Interests

 The authors declare that there is no conflict of interests.

## Ethical Approval

 Ethical eligibility for this research was approved by the Health Research Ethics Committee of the Health Polytechnic of the Ministry of Health Medan (No. 01.25 737/KEPK/POLTEKKES KEMENKES MEDAN 2024). In addition, this research was conducted in three health centers, as evidenced by the existence of a certificate of completion in the mentioned centers (No. 712/PKM-MR/X/2024, No. 755/TU/KL/VIII/2024, and No. 999.9.2/962.2/Pusk.SS/IX/2024 for the Mulyorejo Health Center, the Kutalimbaru Health Center, and the Sei Semayang Health Center, Deli Serdang Regency, respectively).

## Funding

 This research was financially supported by the Health Polytechnic, Ministry of Health, Medan.
